# General Induration of the Arterial System

**Published:** 1829-01-01

**Authors:** 


### TOULON HOSPITAL.

### GENERAL INDURATION OF THE ARTERIAL SYSTEM.

ANTHONY BIGAUD, aged 56 years, entered the hospital of Toulon for the treatment of an old ulcer of the left leg, which leg was covered with varicose veins. The foot had been amputated at Smyrna, in consequence of a viper-bite, some time previously. He also complained of pain in the *left* foot, the little toe of which was observed to be swelled, cold, and of a bluish colour. This man, who was naturally robust and vigorous, had been worn down by long privations and profound chagrin. His answers and his countenance indicated a deep melancholy—he had little appetite—sleep short and interrupted—pulse large, slow, and irregular—the heart beat over a very large surface. The gangrene extended rapidly, and was considered as the consequence of the disease of the heart, which was quite unequivocal. As mortification destroyed the different parts they were removed. The pain was insupportable—the pulse became slower and slower—and the arteries offered such a degree of resistance to pressure as led to the conviction that they were ossified.

On dissection, the heart attracted great attention. It was extremely enlarged; and there were several white patches on its surface. The left ventricle offered as much resistance to the scalpel as thick and dried parchment The origin of the aorta, as well as the aortic valves, were completely cartilaginous—and this was the case with the whole of the aorta, the iliac and crural arteries, and their branches. "Il en était de même de la crosse de l'aorte, de l'aorte pectorale et abdominale, des artères iliaques primitives, de la crurale et de ses distributions." There were several portions of these various arteries *ossified*, besides the whole being cartilaginous. "The arteries of the upper extremities were in a similarly indurated condition."—EPHEMERIDES DE MONTPELLIER, 1828.

It would be difficult for those who advocate the doctrine, that the dilatation and contraction of arteries is *essentially* necessary, at all times and under all circumstances, for the circulation of the blood, to demonstrate how the said circulation was carried on in the above subject. We have never denied that the elasticity, the contractility, and the dilatability of arteries were *necessary* for the well-being of the circulation, taking all its exigencies into the account. But we have never seen any anatomical fact or physiological reasoning to convince us that the dilatation and contraction of arteries were *essentially* necessary in quiet and undisturbed circulation. The above case, and several others on record, prove that life may be carried on when the elasticity and contractility of the whole arterial system is nullified.

---

